# Corneal Sub-Basal Nerve Changes in Patients with Herpetic Keratitis during Acute Phase and after 6 Months

**DOI:** 10.3390/medicina55050214

**Published:** 2019-05-27

**Authors:** Vilija Danileviciene, Reda Zemaitiene, Vilte Marija Gintauskiene, Irena Nedzelskiene, Dalia Zaliuniene

**Affiliations:** 1 Department of Ophthalmology, Medical Academy, Lithuanian University of Health Sciences, Lt-50009 Kaunas, Lithuania; reda.zemaitiene@kaunoklinikos.lt (R.Z.); daliazal@yahoo.com (D.Z.); 2 Department of Immunology and Allergology, Medical Academy, Lithuanian University of Health Sciences, Lt-50009 Kaunas, Lithuania; gintauskiene@yahoo.com; 3 Department of Dental and Oral Pathology, Medical Academy, Lithuanian University of Health Sciences, Kaunas, Lt-50009 Lithuania; Irena.Nedzelskiene@lsmuni.lt

**Keywords:** aesthesiometry, confocal microscopy, sub-basal nerves, herpes simplex virus, keratitis

## Abstract

*Background and objectives:* The purpose of this study was to describe corneal sensitivity and the morphological changes of sub-basal corneal nerves using in vivo laser scanning confocal microscopy (LSCM) in herpes simplex virus (HSV) keratitis-affected eyes, and to compare with both contralateral eyes and with the eyes of patients with a previous history of herpes labialis but no history of herpetic eye disease, and with healthy patients with no history of any HSV diseases, during the acute phase of the disease and after six months. *Materials and Methods:* A prospective clinical study included 269 patients. All of them underwent a complete ophthalmological examination, Cochet-Bonnet aesthesiometry and LSCM within the central 5 mm of the cornea. After six months, all the patients with herpetic eye disease underwent the same examination. Serology tests of the serum to detect HSV 1/2 IgG and IgM were performed. *Results:* HSV-affected eyes compared with contralateral eyes, herpes labialis and healthy control group eyes demonstrated a significant decrease in corneal sensitivity, corneal nerve fibre density, corneal nerve branch density, corneal nerve fibre length and corneal nerve total branch density (*p* < 0.05). During follow up after six months, corneal sensitivity and sub-basal nerve parameters had increased but did not reach the parameters of contralateral eyes (*p* < 0.05). Previous herpes labialis did not influence corneal sensitivity and was not a risk factor for herpetic eye disease. *Conclusions:* Corneal sensitivity and sub-basal nerve changes in HSV-affected eyes revealed a significant decrease compared with contralateral eyes, and with the eyes of patients with a previous history of herpes labialis, and of healthy controls. Following six months, corneal sensitivity and sub-basal nerve parameters increased; however, they did not reach the parameters of contralateral eyes and the eyes of healthy controls. The best recovery of corneal sensitivity was seen in patients with epithelial keratitis. Herpes labialis was not a risk factor for herpetic eye disease.

## 1. Introduction

Herpes simplex virus type 1 (HSV-1) causes herpes labialis and herpetic eye disease, which is a common condition that usually develops as an epithelial, stromal, or endothelial keratitis or uveitis. The disease is a major cause of corneal blindness. The incidence of HSV keratitis is approximately 1.5 million worldwide, including 40,000 new cases of related blindness each year [1,2,3]. In most cases, HSV keratitis/uveitis manifests as a unilateral disease and tends to recur. After the first episode of infection during the first year, the disease recurs in 10% of patients, within 20 years, in 60% of patients [4]. 

Herpes labialis manifests in about 40% of HSV-1 infected humans. For 1/3 of them, clinical manifestation recurs and for half of them it recurs at least two times per year [4].

Herpetic keratitis often presents with decreased corneal sensitivity, which is associated with sensory nerve fibre and branch density changes in sub-basal corneal layer [5,6,7]. Recurrences of the disease may result in corneal scarring, thinning and neovascularisation, more severe changes of corneal nerves and hypesthesia, which poses a major risk in the form of neurotrophic keratopathy due to the loss of the blink reflex, and a reduced influence of sensory nerves to normal corneal physiology [8]. 

Corneal nerves tend to regenerate, but unfortunately, despite significant nerve regeneration, corneal nerves do not fully recover [6,9]. Normal cornea has sensory nerves that enter the stroma, branches, form the subepithelial plexus and extend the terminis into the epithelium. The nerves from the infected cornea hyperinnervate the corneal stroma but do not form a plexus at the subepithelium layer or extend to the epithelium, leaving the cornea less sensitive. Hyperinnervation of corneal stroma by sympathetic nerves determines the severity of HSV keratitis, the disturbance of the sensory nerve plexus, and the regeneration of epithelial nerve endings, which was demonstrated in mouse model [10].

LSCM is a non-invasive method of examining the living human cornea in healthy and pathological sites at the cellular level, detecting epithelial defects, stromal oedema and infiltration, and keratic precipitates [11,12]. Corneal sub-basal nerves, which are reduced due to HSV keratitis, can also be clearly visible by LSCM. This method is rapid, non-invasive and precise, with good interobserver variability [13]. 

Our research constitutes a longitudinal study with the main purpose to describe and correlate data of corneal sensitivity and sub-basal nerve changes in patients with herpetic eye disease and compare with contralateral, clinically unaffected eyes, and with the eyes of patients with previous history of herpes labialis and of healthy subjects during acute phase of the disease and after six months.

## 2. Materials and Methods

A prospective clinical study included 269 patients (170 women (63.2%) 99 men (36.8%), mean age 59.0 ± 8.5 years, range 28.1–84.8). All patients were divided into three groups: 79 patients (38 women (58.1%), 41 men (51.9%), mean age 59.5 ± 9.0 years, range 28.1–76.0) with active unilateral herpetic eye disease, according to the clinical history and clinical examination, 101 healthy patients (72 women (71.3%), 29 men (28.7%), mean age 58.9 ± 8.5 years, range 43.0–84.8) with previous history of herpes labialis, but no history of herpetic eye disease, and 89 patients (60 women (67.4%), 29 men (32.6%), mean age 58.5 ± 7.9 years, range 44.0–80.3) with no history of any HSV diseases. Patients with previous history of other ocular infection, trauma, contact lens wearing, diabetes mellitus, glaucoma, and previous intraocular or refractive surgery were excluded from the study.

The study was approved by the Kaunas Regional Biomedical Research Ethics Committee 2015-07-09 No. BE-2-26 and 2017-01-26 No. P1-BE-2-26/2015. Written informed consent was obtained from all subjects who participated in the study. 

Epithelial keratitis was diagnosed in patients with characteristic multiple epithelial blisters filled with fluid, isolated or merged dendritic infiltrates. Herpes stromal keratitis was diagnosed in patients who had stromal opacities or a destruction of the corneal stroma, ulcerative infiltrates and/or neovascularization. Patients who presented with characteristic swelling of the central part of stroma with the ring infiltrate and precipitate on corneal endothelium had endothelial keratitis. Patients with unilateral keratic precipitates, high intraocular pressure and patchy iris atrophy had anterior herpetic uveitis. 

Both eyes of all patients underwent a complete ophthalmological examination, Cochet-Bonnet aesthesiometry (Cochet-Bonnet; Luneau Ophthalmologie, Chartres, France) within the central 5 mm of the cornea, and LSCM (Heidelberg Retina Tomograph 3 with the Rostock Cornea Module, Heidelberg Engineering GmbH, Dossenheim, Germany) within the central 5 mm of the cornea. Images of all layers of the cornea were obtained, and a special attention was paid to the morphology of the corneal sub-basal nerves. 

The most representative three images of the sub-basal nerve plexus were selected for the analysis of each eye. Sub-basal nerves were categorised as main nerves, branches, which branched from the main nerves, and the total nerves—main nerves and nerve branches.

The corneal nerves analysis was performed using automated Corneal Nerve Fibre Analyser ACCMetrics V.2. The software automatically extracts and quantifies nerve fibre metrics in images obtained using the Heidelberg HRT III corneal confocal microscope with 384 × 384 pixels and the field of view of 400 × 400 μm^2^ (resolution: 400/384 = 1.0417 μm). It quantifies the corneal nerve fibre length (CNFL)—the total length of nerves mm/mm^2^, nerve fibre density (CNFD)—the number of fibres/mm^2^, nerve branch density (CNBD)—the number of branch points on the main fibres/mm^2^, and nerve fibre total branch density (CTBD)—the total number of branch points/mm^2^ [14,15,16,17,18,19,20].

After six months from the first clinical examination, the same ophthalmological investigation was repeated, and the data were compared with the primary test results.

For all patients, serology tests of the serum to detect HSV 1/2 IgG and IgM using Virion Serion ELISA classic kit were performed (sensitivity and specificity >98%). Negative results were when IgG or IgM concentrations were <20 U/mL, possible 20–30 U/mL, positive >30 U/mL. IgG positive result were divided into two groups: I—when concentration was less than 10,000 U/mL, II—when concentration was more than 10,000 U/mL.

The statistical analysis was performed with SPSS 23 programme. All parametric data were expressed as the mean and standard deviation. Kolmogorov-Smirnov test was used for determination of quantitative data distribution. The results were analysed by Kruskal-Wallis and Mann-Whitney tests. The Kruskal-Wallis test was applied to compare the scores for more than two independent groups and the Mann-Whitney, for the scores of two independent groups. Differences between dependent variables were analysed by the Wilcoxon signed-rank test. The Pearson correlation coefficient was calculated to determine the relationship between nerve density parameters and aesthesiometry results in HSV affected eyes. 

For comparing the frequencies of qualitative variables, χ^2^ tests were used. In order to assess minimally false negative and minimally false positive results with greatest accuracy, the method of ROC (receiver operating characteristics) curve was used. Logistic regression analysis was performed to determine the odds ratio predictive value. Differences were considered statistically significant, when *p* values < 0.05.

## 3. Results

In total, 79 patients (42 OD, 27 OS (*p* > 0.05)) with active herpetic eye disease, including epithelial (*n* = 48), stromal (*n* = 15), or endothelial keratitis (*n* = 16), 101 patients with previous history of herpes labialis, and 89 patients with no history of HSV diseases were analysed and compared. Demographic data of all groups and subgroups are presented in Table 1.

The mean time from the beginning of herpetic eye disease until the patient was examined at our hospital was 50.0 ± 64.5 (Median 23.0 (min—3, max—344)) days. 54(68.4%) patients had already had the antiviral treatment for 15.3 ± 36.7 days (Median 4.0 (min—0, max—239)), but this did not affect the presented results of corneal sensitivity, intraocular pressure and corneal sub-basal nerve fiber parameters (*p* > 0.05).

A total of 36 (45.6%) patients with herpetic eye disease previously had herpes labialis. For 41.7% of patients, herpes labialis presented when they were 20–30 years old. We did not find previous herpes labialis and its recurrence quantity to be a risk factor for herpetic eye disease, but comparing those who contracted the disease, there were more chances for herpetic eye disease if the patient contracted the first herpes labialis during childhood OR (95% CI), 4.524 (1.448–14.135) (*p* < 0.05). Herpes did not influence corneal sensitivity either (*p* > 0.05).

It was found that 92.3% of all subjects had negative HSV IgM and for 7.7% it was possible or positive. However, IgG was negative only for 1.4% of all subjects: 81.4% had less than 10,000 U/mL, 17.2% more than 10,000 U/mL. The differences in the results of IgG between the groups were not statistically significant (χ^2^ = 5.943, df = 4, *p* = 0.203). The only significant result when comparing IgM, was fewer patients with negative IgM in the herpetic eye disease group compared with the herpes labialis and healthy control groups (IgM < 20 U/mL 86.3% vs. 94.7% vs. 95.4% subjects) (*p* < 0.05).

**Figure 1:**
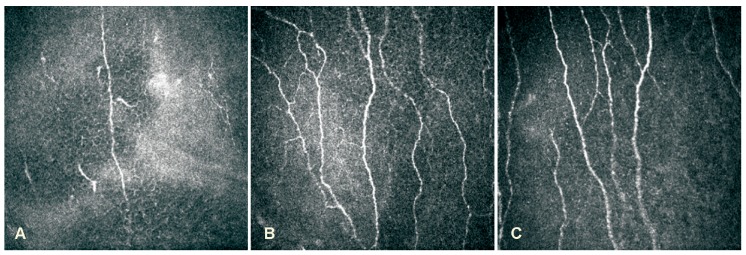
LSCM images of corneal sub-basal nerve plexus: (**A**)–HSV keratitis with severe sensation loss; (**B**)–Herpes labialis patient; (**C**)–Healthy person.

HSVaffected eyes showed a reduction in the mean corneal sensitivity and sub-basal nerve parameters and an increase in the mean intraocular pressure when compared with contralateral eyes, herpes labialis and healthy control group eyes (*p* < 0.05). The mean corneal sensitivity and sub-basal nerve parameters of contralateral eyes were also different comparing with herpes labialis patients and healthy controls (*p* < 0.05). There was no difference between herpes labialis and healthy control group eyes (*p* > 0.05). Data of all groups and subgroups are presented in Table 2. LSCM images of corneal sub-basal nerve plexus are demonstrated in Figure 1.

Subgroup analysis of herpetic eye disease demonstrated that the eyes with epithelial, stromal, endothelial keratitis had a reduced corneal sensitivity and sub-basal nerve plexus parameters, as compared with the contralateral eye parameters, herpes labialis, and healthy control group eyes (*p* < 0.05). But there was no difference of corneal nerve and other parameters between herpetic eye disease subgroups (*p* > 0.05), except the intraocular pressure, which was higher in the endothelial keratitis subgroup (*p* = 0.01).

CNFL and CTBD in herpetic eyes were lower with age and CNFL was lower with increased tearing (*p* < 0.05). With decreased CNFL and CNBD, corneal sensitivity was lower (*p* < 0.05). Intraocular pressure didn’t influence any of the parameters (*p* > 0.05). In healthy controls, corneal sensitivity was lower and the intraocular pressure was higher with age (*p* < 0.05).

Based on receiver operating characteristic (ROC) analysis herpetic keratitis could be suspected when CNFD and CNBD is less than 3.13 n/mm^2^, CNFL less than 9.92 mm/mm^2^, and CTBD less than 15.62 n/mm^2^ in unknown ethiology keratitis eye, and CNFD less than 15.62 n/mm^2^, CNBD less than 16.15 n/mm^2^, CNFL less than 10.07 mm/mm^2^, and CTBD less than 37.5 n/mm^2^ in the contralateral eye. The detailed ROC analysis is presented in Table 3.

**Figure 2:**
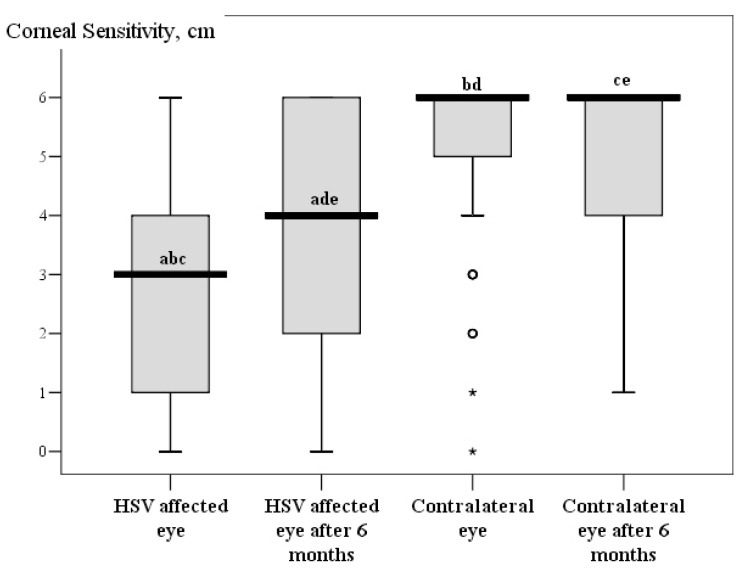
Corneal sensitivity parameters Boxplot during the first visit and follow up. ^a,b,c,d,e^ *p* < 0.001 by Wilcoxon test.

**Figure 3:**
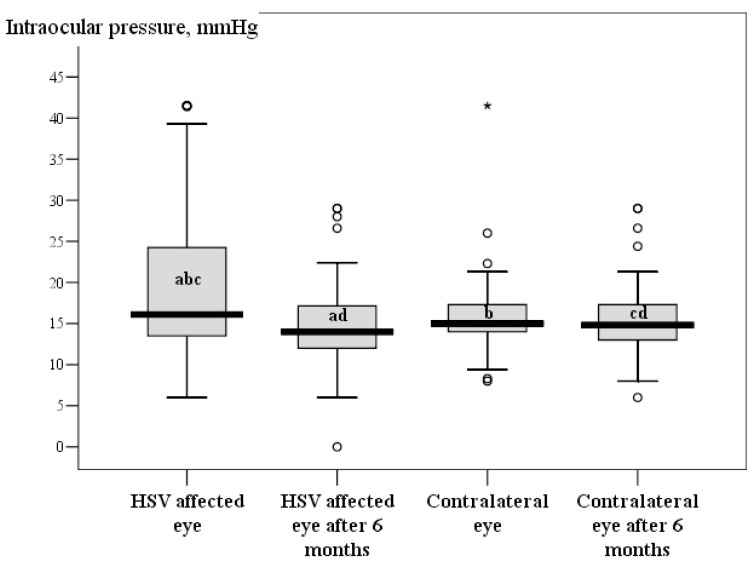
Intraocular pressure parameters Boxplot during the first visit and follow up. ^a,b,c,d^ *p* < 0.05 by Wilcoxon test.

After six months, all of the examinations were performed again on patients with herpetic eye disease and compared with the primary examination data. During the follow up, corneal sensitivity increased in HSV affected eyes but did not reach the contralateral eye or herpes labialis and healthy controls (*p* < 0.05) Changes in corneal sensitivity and intraocular pressure are demonstrated in Figure 2 and Figure 3. All of the corneal sub-basal nerve parameters increased during the follow up in HSV affected patients, but only CNFD and CNBD were statistically significant in HSV affected eyes (*p* < 0.05). However, the parameters of HSV-affected eyes did not reach the parameters of contralateral eyes and of the herpes labialis and healthy control groups (*p* < 0.05). Further parameters of contralateral eyes did not reach those of herpes labialis or the healthy controls’ eyes (*p* < 0.05). Data of sub-basal nerve parameter changes are presented in Table 4.

## 4. Discussion

Cornea is the most densely innervated structure in the human body. Corneal nerves are responsible for reflex, sensory, and trophic functions and play an important role in blinking, tear production and secretion, the regulation of epithelial integrity, proliferation, and wound healing [21,22,23].

Several infections, such as herpetic eye disease, result in corneal nerve damage which leads to the formation of neurotrophic keratopathy with progressive cornea epithelial cell loss, oedema, and blindness. Although the mechanism of this trophic role is not fully understood, in vivo confocal microscopy has provided new knowledge of corneal nerves and is a gold standard for the examination and imaging of corneal cellular structure [24].

During the study we compared the parameters of intraocular pressure, corneal sensitivity and sub-basal nerves in HSV keratitis patients. The novelty of the study is to compare the results during the primary visit and after six months for HSV patients and to collate them not only with healthy controls, but also with the subjects who had a previous history of herpes labialis but no history of herpetic eye disease. Previously, we conducted another study where we compared corneal sensitivity and sub-basal nerve changes in HSV keratitis patients. The results are soon to be published in International Journal of Ophthalmology. In the first study we included only 30 patients and there was no healthy controls group. For more accurate results we decided to expand the study to include more HSV patients and two control groups. Here we present the results that confirm and complement the results of our initial study.

During the study we found a decrease of sub-basal nerve parameters in HSV affected eyes which correlated with corneal sensitivity. Moreover, the parameters of contralateral eyes were also lower when comparing with herpes labialis and healthy controls. Similar results have been previously reported in several studies [5,7,25]. Moein et al. did not find corneal sensitivity change in the contralateral eye during the acute phase of herpetic eye disease [6]. Nevertheless, based on most of the study’s findings, HSV corneal disease could potentially lead to a loss of corneal innervation not only in HSV affected, but also in contralateral eyes [7,25]. Herpetic eye disease is usually unilateral. The incidence of bilateral HSV keratitis has been reported to be from 1.3% to 12% [26]. The exact mechanisms of contralateral eye changes remain unclear. It could be explained by the central nervous system-mediated contralateral effects, where central nervous system pathways are responsible for affecting contralateral undamaged neurons [27,28]. On the other hand, this response might be caused by a viral expansion to the contralateral side, with or without clinical symptoms [29]. During the virus reactivation, HSV-1 has been found in clinically unaffected corneas in small amounts [30]. The virus reactivation might occur not only in the trigeminal ganglion but also in the cornea. This would explain the absence of clinically recognizable disease in the fellow eye and the differences in sub-basal nerve parameters and cornea sensitivity in herpes keratitis, contralateral, and healthy patients’ eyes [31]. Alternatively, the virus might travel from the trigeminal ganglia between nerve anastomosis to the contralateral mesencephalic trigeminal nucleus. This would allow primary trigeminal fibres to cross the pontine tegmentum to reach the contralateral principal nucleus and to cause contralateral damage to the distal nerve plexus without contralateral clinical manifestations [25,32]. In addition, biochemical mechanisms may also play a role by releasing inflammatory mediators from neurons [27,28,33].

However, during the study we did not notice a significant difference in corneal sensitivity and sub-basal nerve parameters between herpes labialis and healthy controls. Also, we did not notice any correlation between sub-basal nerve alterations and the number of herpetic keratitis recurrences as it has been presented in a study by Hamrah et al. [5].

Based on our results the intraocular pressure was higher in the eyes with the endothelial type of keratitis. Corneal nerve parameters and corneal sensitivity was higher and closer to contralateral eyes in patients with endothelial type of keratitis comparing with other subgroups of herpetic keratitis, but the difference was not statistically significant. Similar results of corneal nerve parameters and corneal sensitivity with statistical confirmation have been submitted in the study by Nagasato et al. [7]. During endothelial HSV keratitis, the virus invades endothelial cells with a reduction of endothelium cell density and pleomorphism, likely damaging corneal sub-basal nerves less. In vivo confocal microscopy in eyes affected by HSV endotheliitis identifies a decrease in endothelial cell density, infiltration of inflammatory cells and enlargement of intercellular gaps [34].

Based on our study, only half of the patients with herpetic eye disease had previously had herpes labialis, most of them 1–2 times per year. We first established that previous herpes labialis and its recurrence quantity was not a risk factor for herpetic eye disease and did not influence corneal sensitivity, but comparing those who contracted the disease, there were more chances for herpetic eye disease if the patient got the first herpes labialis during childhood.

Serological methods are used widely for the determination of HSV IgG and IgM antibodies in virological laboratories. Sensitivity is between 89.1% and 98.0% and specificity from 82.8% to 100% [35]. About 90 percent of the population older than 50 years is infected with HSV. Only 4.3% of our study’s healthy controls had negative IgG rate. However, we did not find any correlations between IgM or IgG rates and corneal sensitivity or corneal sub-basal nerve parameters, intraocular pressure, herpes labialis or herpetic keratitis reoccurrence and frequencies.

In patients who have or have had HSV keratitis, corneal sensitivity becomes lower [36]. It has been known that corneal sensitivity and sub-basal nerves are also impaired by age [37,38]. Parissi et al. determined a mean nerve density 20.1 ± 4.5 mm/mm^2^ for 46–60 years old healthy controls and 16.7 ± 5.5 mm/mm^2^ for those older than 60 [39]. Our study results of healthy controls showed that with age corneal nerve fibre length and corneal nerve total branch density decreases and corneal sensitivity becomes lower and irreversible. According to the age of the subjects we found that in HSV affected and contralateral eyes corneal sensitivity and the parameters of corneal sub-basal nerves had increased after 6 months, however, the parameters of the affected eyes were not the same as those of contralateral, herpes labialis or healthy controls’ eyes. The corneal nerve fibre density and corneal nerve fibre length of contralateral eyes did not reach that of herpes labialis and healthy controls’ eyes. The best recovery of corneal nerve fibre density after 6 months was seen in patients with first time HSV keratitis. The best recovery of corneal nerve total branch density and corneal sensitivity was in patients with epithelial keratitis. Moein et al. have found similar results of corneal sub-basal nerve damage and regeneration, but they have not observed a corneal sensitivity change in HSV keratitis affected eyes or in contralateral eyes during the follow up [6]. Although the regeneration of corneal nerves is still not fully explained, it has been shown that the process is multifactorial [40]. One of the components of the corneal nerve regeneration process is axonal growth cone guidance molecules—semaphorins, which promote axonal outgrowth and regeneration and concentrate inflammatory cells [9,41]. During corneal damage, an increased immune response correlates with decreased innervations. Cruzat et al. have demonstrated a correlation between the increase of dendritic cell density and the decrease in sub-basal corneal nerves in patients with infection keratitis, suggesting a connection between the corneal immune response and nerve alterations [42]. Nerve regeneration and inflammation processes are also related in tissue repair. Nerve injury initiates inflammation, injured corneal stromal fibroblasts release semaphorins, neurotrophins, immune regulatory factors and matricellular proteins and play a role in nerve regeneration growth, differentiation and wound healing [43].

To conclude our results, we recommend suspecting herpetic keratitis when CNFD and CNBD is less than 3.13 n/mm^2^, CNFL less than 9.92 mm/mm^2^, and CTBD less than 15.62 n/mm^2^ in unknown ethiology keratitis eye and CNFD less than 15.62 n/mm^2^, CNBD less than 16.15 n/mm^2^, CNFL less than 10.07 mm/mm^2^, and CTBD less than 37.5 n/mm^2^ in the contralateral eye.

A limitation of our study may be related to the evaluation of only the central part of the cornea, Cochet-Bonnet aesthesiometer, which only measures mechanical nociceptors, and LSCM testing method, which is a contact diagnostic tool and causes an ocular discomfort to a patient leading to increasing eye movements that sometimes blur the images.

The results of our study could be informative for clinical practice as a part of diagnostic procedures which help to diagnose herpetic eye disease, and to evaluate the severity of the infection. However, a more detailed evaluation of corneal lessions, including the counting of dendritic cells and endothelium cell density is required to produce more accurate conclusions and recommendations.

## 5. Conclusions

In vivo laser scanning confocal microscopy constitutes an effective tool for diagnosing HSV keratitis and detecting the morphological changes of sub-basal corneal nerves.

Corneal sensitivity and sub-basal nerve changes in HSV affected eyes revealed a significant decrease compared with contralateral eyes and the eyes of patients with previous history of herpes labialis and of healthy patients. There was no significant difference in the parameter between keratitis subgroups, or between healthy controls and herpes labialis patients. During the following six months, corneal sensitivity and sub–basal nerve fibre parameters had increased, however, they did not reach the clinically unaffected eyes and healthy controls. The best recovery of corneal nerve fibre density after six months was seen in patients presenting for the first time with HSV keratitis. The best recovery of corneal sensitivity was in patients with epithelial keratitis.

## Figures and Tables

**Table 1:** Demographic data of patients with HSV keratitis and control groups.

	HSV Keratitis			Total	Herpes Labialis Group	Healthy Controls
	Epithelial	Stromal	Endothelial			
Patients, *n*	48	15	16	79	101	89
Age (years) (mean ± SD)	61.0 ± 8.9	55.7 ± 8.1	57.2 ± 11.4	59.9 ± 9.0	58.9 ± 8.5	58.5 ± 7.9
Age range (years)	28.1–75.9	38.3–66.3	28.6–70.0	28.1–76.0	43–84.8	44–80.3
Sex, *n*(female/male)	29/19	4/11	5/8	38/41	72/29	60/29

There was no age difference between groups (*p* > 0.05).

**Table 2:** Quantitative analysis of corneal sensitivity, intraocular pressure and sub-basal nerve fibre parameters in HSV keratitis subgroups, contralateral eyes and control groups.

	HSV Keratitis			Total *n* = 79	Contralateral Eye *n* = 79	Herpes Labialis Group *n* = 101	Healthy Controls *n* = 89
	Epithelial *n* = 48	Stromal *n* = 15	Endothelial *n* = 16				
CNFD n/mm^2^	2.2 ± 5.4 ^a^	1.7 ± 3.7	5.3 ± 8.4 ^a^	2.6 ± 5.8 ^a,b,c^	9.9 ± 7.6 ^a,d,e^	19.1 ± 12.2 ^b,d^	15.8 ± 10.8 ^c,e^
CNBD n/mm^2^	2.0 ± 5.5 ^b^	0.8 ± 2.2 ^c^	8.2 ± 13.1 ^b,c^	2.8 ± 7.3 ^a,b,c^	11.7 ± 13.5 ^a,d,e^	22.0 ± 20.7 ^b,d^	21.3 ± 24.2 ^c,e^
CNFL mm/mm^2^	6.0 ± 4.2	6.2 ± 3.7	8.4 ± 5.3	6.5 ± 4.3 ^a,b,c^	9.6 ± 6.3 ^a,d,e^	12.8 ± 5.2 ^b,d^	11.9 ± 4.9 ^c,e^
CTBD n/mm^2^	19.8 ± 22.6	21.3 ± 18.4	22.6 ± 25.5	20.6 ± 22.1 ^a,b^	25.2 ± 20.6 ^c,d^	38.4 ± 31.2 ^a,c^	38.9 ± 34.0 ^b,d^
Corneal sensitivity, cm	2.8 ± 2.0	2.2 ± 1.6 ^d^	3.7 ± 1.3 ^d^	2.8 ± 1.9 ^a,b,c^	5.1 ± 1.4 ^a,d,e^	5.7 ± 0.6 ^b,d^	5.7 ± 0.6 ^c,e^
Intraocular pressure mmHg	17.1 ± 6.8 ^e^	19.5 ± 9.1	25.4 ± 10.6 ^e^	19.0 ± 8.5 ^a,b,c^	15.5 ± 4.3 ^a,d,e^	14.1 ± 3.0 ^b,d^	14.9 ± 3.6 ^c,e^
	^a,b,c,d,e^*p* < 0.05, by Mann-Whitney test						

Values reported as mean ± SD. CNFD, corneal nerve fibre density; CNBD, corneal nerve branch density; CNFL, corneal nerve fibre length; CTBD, corneal nerve total branch density.

**Table 3:** Distribution of predicted values and characteristics of CNFD, CNBD, CNFL, and CTBD parameters for ROC test by groups of subjects.

Parameter/Threshold Value	Area under ROC Curve (%)	Sensitivity/Specificity (%)	HSV Affected Patients/Herpes Labialis and Healthy Controls (%)	*p*-Value	OR of HSV Affected Patients (95% CI)
CNFD herpetic eye <3.13	87.5	78.5 91.1	78.5 8.9	<0.001	37.114 (17.847–77.184)
CNFD healthy eye <15.62	70.0	80.6 50.5	80.6 49.5	<0.001	4.242 (2.173–8.282)
CNBD herpetic eye <3.13	80.0	82.3 76.3	82.3z 23.7	<0.001	14.96 (7.676–29.158)
CNBD healthy eye <16.15	62.2	73.1 50.5	73.1 49.5	0.001	2.78 (1.51–5.119)
CNFL herpetic eye <9.92	80.6	83.5 65.3	83.5 34.7	<0.001	9.538 (4.903–18.557)
CNFL healthy eye <10.07	68.9	67.2 63.7	67.2 36.3	<0.001	3.587 (1.99–6.467)
CTBD herpetic eye <15.62	67.1	58.2 72.6	58.2 27.4	<0.001	3.699 (2.136–6.407)
CTBD healthy eye <37.5	61.3	80.6 41.6	80.6 58.4	0.001	2.956 (1.512–5.782)

OR—odds ratio; CI—confidence interval.

**Table 4:** Quantitative analysis of corneal sub-basal nerve changes during the first visit and follow up.

	HSV Affected Eye *n* = 79		Contralateral Eye *n* = 79		Herpes Labialis Group *n* = 101	Healthy Controls *n* = 89
	First Visit	Follow up	First Visit	Follow up		
CNFD n/mm^2^	2.5 ± 5.7 ^a,b1^	6.5 ± 7.0 ^a,b6,c,e^	9.9 ± 7.6 ^a,b1^	12.6 ± 10.3 ^a,b6,d^	19.1 ± 12.2 ^c,d^	15.8 ± 10.8 ^e^
CNBD n/mm^2^	2.7 ± 7.2 ^a,b1^	8.1 ± 10.7 ^a,b6,c,e^	11.7 ± 13.5 ^a,b1^	15.2 ± 16.2 ^a,b6^	22.0 ± 20.7 ^c^	21.3 ± 24.2 ^e^
CNFL mm/mm^2^	6.3 ± 4.3 ^b1^	7.3 ± 4.3 ^b6,c,e^	9.6 ± 6.3 ^a,b1,d,f^	9.7 ± 4.2 ^a,b6^	12.8 ± 5.2 ^c,d^	11.9 ± 4.9 ^e,f^
CTBD n/mm^2^	20.1 ± 21.9	22.2 ± 18.5 ^c,e^	25.2 ± 20.6 ^a^	29.7 ± 25.6 ^a^	38.4 ± 31.2 ^c^	38.9 ± 34 ^e^
	^a,b1,b6,c,d,e,f^*p* < 0.05					

^a^—*p* value between parameters of the first visit and follow up; ^b1^—*p* value between parameters of HSV affected and contralateral eyes during the first visit; ^b6^—*p* value between parameters of HSV affected and contralateral eyes during follow up by Wilcoxon test; ^c^—*p* value between parameters of HSV affected eye during follow up and herpes labialis group; ^d^—*p* value between parameters of contralateral eye during follow up and herpes labialis group; ^e^—*p* value between parameters of HSV affected eye during follow up and healthy controls; ^f^—*p* value between parameters of contralateral eye during follow up and healthy controls by the Mann-Whitney test.
